# Characterization of a *Plasmodium falciparum* Orthologue of the Yeast Ubiquinone-Binding Protein, Coq10p

**DOI:** 10.1371/journal.pone.0152197

**Published:** 2016-03-25

**Authors:** Bethany J. Jenkins, Thomas M. Daly, Joanne M. Morrisey, Michael W. Mather, Akhil B. Vaidya, Lawrence W. Bergman

**Affiliations:** Center for Molecular Parasitology, Drexel University College of Medicine, Philadelphia, PA, United States of America; Université Pierre et Marie Curie, FRANCE

## Abstract

Coenzyme Q (CoQ, ubiquinone) is a central electron carrier in mitochondrial respiration. CoQ is synthesized through multiple steps involving a number of different enzymes. The prevailing view that the CoQ used in respiration exists as a free pool that diffuses throughout the mitochondrial inner membrane bilayer has recently been challenged. In the yeast *Saccharomyces cerevisiae*, deletion of the gene encoding Coq10p results in respiration deficiency without inhibiting the synthesis of CoQ, suggesting that the Coq10 protein is critical for the delivery of CoQ to the site(s) of respiration. The precise mechanism by which this is achieved remains unknown at present. We have identified a *Plasmodium* orthologue of Coq10 (PfCoq10), which is predominantly expressed in trophozoite-stage parasites, and localizes to the parasite mitochondrion. Expression of PfCoq10 in the *S*. *cerevisiae coq10* deletion strain restored the capability of the yeast to grow on respiratory substrates, suggesting a remarkable functional conservation of this protein over a vast evolutionary distance, and despite a relatively low level of amino acid sequence identity. As the antimalarial drug atovaquone acts as a competitive inhibitor of CoQ, we assessed whether over-expression of PfCoq10 altered the atovaquone sensitivity in parasites and in yeast mitochondria, but found no alteration of its activity.

## Introduction

Ubiquinone [CoQ] plays an essential role in cellular respiration that is conserved from prokaryotes to eukaryotes, serving as an electron acceptor/donor for several mitochondrial respiratory complexes and dehydrogenases. It is composed of a benzoquinone ring and a polyprenyl tail, the length of which varies between organisms. In *Saccharomyces cerevisiae*, the enzymes involved in the synthesis of CoQ from these precursors have been identified by studies characterizing a series of respiration-deficient mutants, with lesions in genes now termed *COQ1*-*CQQ9* [1]. In addition to their inability to grow on a non-fermentable carbon source, these mutants accumulated metabolic intermediates of CoQ synthesis [2]. A tenth respiration-deficient mutant, in which CoQ synthesis was inefficient but not entirely inhibited, led to the discovery of *COQ10* [3, 4]. Because of this unique phenotype, it was hypothesized that Coq10p may act as a chaperone for the transport of CoQ within the mitochondria from its site of synthesis to the respiratory complexes [3, 4]. The Coq10 protein is characterized by a lipophilic START (Steroidogenic Acute Regulatory-related lipid Transfer) domain, with conserved orthologues found from bacteria to humans [3]. Coq10p has been shown to bind ubiquinone in its hydrophobic pocket [5], but does not appear to be in a complex with the other CoQ synthesis enzymes [6].

*Plasmodium falciparum* synthesizes CoQ with tail lengths of 8 and 9 isoprenyl units [7],and serves as the electron acceptor for five mitochondrially located dehydrogenases [8]. Re-oxidation of CoQH_2_ (reduced CoQ) occurs at the respiratory complex III, the ubiquinone-cytochrome *c* oxidoreductase, also known as the cytochrome *bc*_1_ complex [9]. The *bc*_1_ complex is a validated target for the antimalarial drug atovaquone, which inhibits the enzyme by binding to the CoQH_2_ oxidation site of the complex, thereby blocking the mitochondrial electron transport chain (mtETC) [9]. The essential function of the mtETC in the blood stages appears to be re-oxidation of CoQ to serve dihydroorotate dehydrogenase, a critical enzyme involved in de novo synthesis of pyrimidine precursors. Expression of a cytosolic ubiquinone-independent yeast DHOD renders parasites resistant to atovaquone as well as all mtETC inhibitors [10].

In apicomplexans, the isoprenoid precursor is generated in the relic plastid, termed the apicoplast, and this biosynthesis is an essential function of this organelle [11, 12], while the benzoquinone ring is synthesized via the shikimate pathway [11, 13]. The synthesis pathways of CoQ precursors are divergent from those in mammals and have been suggested as potential drug targets [12, 13]. While several orthologues of *S*. *cerevisiae* Coq1-Coq9 have been identified and their localization to the mitochondrion confirmed [14, 15], much of this pathway remains to be characterized in malaria parasites. Given the divergent origin of its precursors and its essential role in mtETC, CoQ synthesis and regulation in *Plasmodium* could offer promising targets for novel antimalarials.

We have identified PfCoq10, the *P*. *falciparum* orthologue of ScCoq10, and show that expression of this protein in *coq10* null yeast restores cellular respiration, suggesting its ability to bind and transport ubiquinone. PfCoq10 is primarily expressed in trophozoite stage parasites and localizes to the mitochondrion. While ubiquinone has some structural similarity to the antimalarial drug atovaquone, our data suggests that PfCoq10 does not functionally interact with atovaquone.

## Materials and Methods

### *Plasmodium* cell culture and transfection

All transfections were performed in *P*. *falciparum* 3D7attb parasites [16]. Parasites were maintained in RPMI 1640 supplemented with 0.5% Albumax, 15 mM HEPES, 2 g/L sodium bicarbonate, 50 mg/ml gentamycin, and 1 mg/ml hypoxanthine, and maintained at 5% hematocrit. Parasitemia was determined by Giemsa smears. Cultures were synchronized with 2 volumes of 0.3 M alanine buffered with 10 mM HEPES (pH 7.5). For experiments requiring tight synchronization, alanine treatment was performed twice at 8–12 h intervals. Transfections were performed on ring stage parasites at 5% parasitemia. After washing 3 times with cytomix, parasites were suspended in cytomix to 50% hematocrit, mixed with 50–100 μg of plasmid DNA, and electroporated using a Biorad gene pulser (0.31 kV, 960 μFD). All transfections using the 3D7 attB parasites were also co-transfected with an integrase vector and selected with blasticidin (InvivoGen) and G418 (Cellgro) starting at 48 hours post-transfection. Integration of the transfected plasmid at the GLP3 site was confirmed by PCR.

### Western blot

Approximately 2 X10^5^ infected erythrocytes were collected and lysed in 0.5% saponin. The pellet was resuspended in SDS buffer containing 2% β-mercaptoethanol, and separated by SDS-PAGE. After transfer to a nitrocellulose membrane, blots were blocked in 5% milk, and probed with either mouse anti-GFP (1:10,000) or mouse anti- hemagglutinin (HA) epitope (Santa Cruz Biotechnology; 1:10,000 dilution), and subsequently with rabbit or goat anti-mouse IgG conjugated with HRP (secondary 1:1000), and developed on film with SuperSignal West Femto substrate (ThermoFisher Scientific). Plasmodium aldolase was detected using a rabbit anti-aldolase (Abcam) directly conjugated to HRP (1:40,000 dilution).

### Microscopy

Late stage parasites were incubated in 60 nM MitoTracker Red CMXRos (Invitrogen) for 30 min., washed 3X in PBS, and fixed with 4% formaldehyde and 0.0075% glutaraldehyde overnight. Parasites were permeabilized with 0.1% Triton-X 100, reduced with 0.1M glycine, and blocked with 5% fetal bovine serum (FBS). The permeabilized parasites were incubated with mouse anti-HA antibody (Santa Cruz) diluted 1:100 in 5% FBS for at least one hour at room temperature and subsequently for one hour with goat anti-mouse IgG-alexafluor488 (Jackson Immuno) diluted 1:250. The cells were washed briefly with a DAPI (4',6-Diamidino-2-phenylindole) solution, mounted in antifade (Invitrogen), and visualized on an Olympus BX60 microscope. Images were analyzed using Slidebook software.

### Generation of parasite expression vectors

PfCoq10 was amplified from blood stage cDNA using the following primers: PF-10-5AV and PF-10-3BSI (S1 Appendix), digested with AvrII and BsiWI and cloned into the pLN vector, containing either a carboxy-terminal triple HA (3HA) or GFP tag [16]. For expression of PfCoq10 from its native promoter, a 1255 bp fragment corresponding to -1259 to -4 (relative to the ATG start codon) was amplified using primers PF-10-5AP and PF-10-3AV (see S1 Appendix) with ApaI and AvrII restrictions sites and used to replace the Pf calmodulin promoter in the pLN vector.

### Generation of yeast expression vectors

A codon-optimized PfCoq10 gene was synthesized (Genewiz) with a 5’-BamHI site (immediately upstream of the ATG codon) and a 3’-BsiWI site (immediately after the last codon). This was cloned in frame to a 3HA epitope element in the vector pRS426 containing the yeast *TDH1* promotor and a 3’ region from the yeast *CYC1* gene. To remove the mitochondrial localization sequence, the full length codon-optimized gene was subjected to PCR with the primer PF- Δ34B, containing a 5’BamHI site and an ATG codon in combination with a primer that was vector specific (M13-REV primer) (S1 Appendix). The resulting fragment was cloned into the vector described above. The wild type *ScCOQ10* gene, including 887 bp 5’ of the ATG codon was amplified from genomic DNA using primers Sc-5S (containing a SacI site) and Sc3BSI (containing a BsiWI site; see S1 Appendix) and cloned in frame to a 3HA element in the vector pRS416.

### Yeast transformation and complementation studies

A haploid *S*. *cerevisiae* strain (BY4742) containing a null allele of *COQ10* (*coq10*::*Kan*) was obtained from Dr. Santosh Katiyar (Drexel University) from the collection of yeast deletion strains (Research Genetics/Invitrogen). This strain was transformed using the lithium acetate procedure [17] with plasmids expressing the full length *PfCOQ10* gene, the *PfCOQ10-Δ34* gene lacking the presumptive mitochondrial targeting sequence, the wild type *ScCOQ10* gene or the empty vector alone. Transformants were selected by growth in synthetic media lacking uracil and. containing glucose as a carbon source. Individual transformants were grown, collected by centrifugation and resuspended in sterile deionized water. Approximately 10^5^ yeast cells, and subsequent 10-fold serial dilutions were spotted on synthetic media plates lacking uracil and containing either 2% glucose or 3% glycerol as a carbon source. Yeast ferment glucose as a carbon source, whereas glycerol is a nonfermentative carbon source and requires mitochondrial function for yeast to grow. Growth in a nonfermentable carbon source, such as glycerol, depends upon oxidative phosphorylation requiring a significant change in gene expression including induction of genes involved in gluconeogenesis, the glyoxylate cycle, the tricarboxylic acid cycle and active mitochondrial electron transport chain. Thus, yeast lacking its *COQ10* gene are unable to grow with glycerol as the sole carbon source, but are able to do so when complemented with a *COQ10* gene. Plates were incubated at 30°C for 3 days (glucose as carbon source) or 6 days (glycerol as carbon source. To confirm expression of the PfCoq10 proteins, cultures were grown in media lacking uracil with glucose as the carbon source, collected by centrifugation, resuspended in SDS-sample buffer and lysed by vortexing in the presence of glass beads. Aliquots were analyzed by SDS-PAGE and Western blotting performed using a monoclonal mouse anti-HA-HRP conjugate (Santa Cruz; 1:10,000 dilution).

### Isolation of yeast mitochondria and respirometry

Yeast strains (described above) were grown in media (6.7 g/L yeast nitrogen base, 5 g/L casamino acids supplemented with histidine, adenine, tryptophan, and 2% raffinose) to an OD_600_ of 2. The yeast pellet was prepared and mitochondria isolated as previously described [18]. Mitochondrial protein concentrations were measured by Bradford assay (BioRad). Respirometry experiments were performed on a Strathkelvin Instruments Mitocell respirometry system equipped with a model 1302 oxygen electrode. NADH was added at a final concentration of 1 mM to 11 μg of mitochondria in a 75 μl reaction volume. ADP was added to a final concentration of 2.3 mM.

### Hypoxanthine incorporation assay

Parasite growth inhibition by atovaquone was determined using a modified version of the ^3^H-hypoxanthine incorporation assay [19]. *P*. *falciparum* 3D7 parasites in culture were exposed to serial dilutions of atovaquone for 48 hrs. and ^3^H-hypoxanthine for the last 24 hrs. The incorporation of ^3^H-hypoxanthine into parasite nucleic acids was determined by liquid scintillation counting, and the dose-response data (EC_50_) were analyzed using nonlinear regression analysis (Prism GraphPad).

## Results

### PF3D7_0807400 shares homology with *S*. *cerevisiae* Coq10

**Fig 1:**
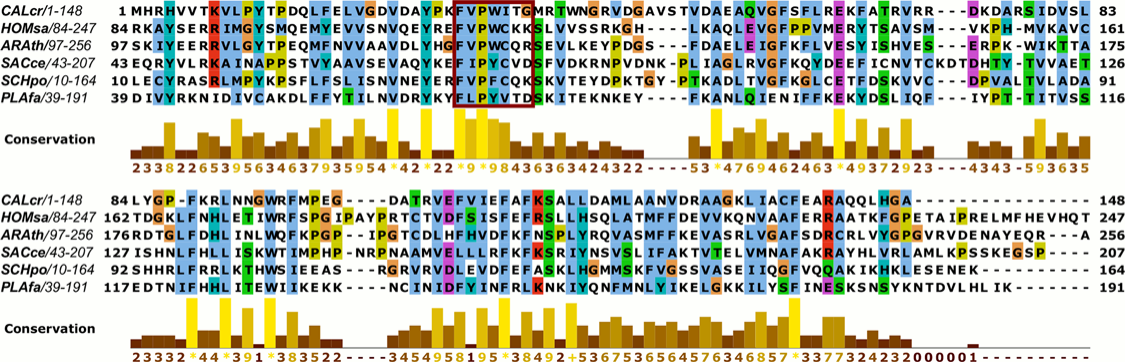
Mulitple sequence alignment of Coq10 ortholoques from selected divergent spp., showing the conservation of ubiquinone-binding residues. A sequence alignment of PF3D7_0807400, *S*. *cerevisiae* Coq10, *S*. *pombe* Coq10p, *Homo sapiens* coenzyme Q-binding protein COQ10 homolog A, *Arabidopsis thaliana* START domain protein AT4G17650, and *Caulobacter crescentus* START domain protein CC1736 (the structure of this bacterial START protein has been determined [21], and it has been shown to bind CoQ [4]) is shown. The red colored box delineates the ubiquinone-binding region identified in *S*. *pombe* [5]. The alignment was generated using MAFFT [22] (L-INS-i method).

We used InterPro [20] to identify START domain-containing proteins in the *Plasmodium* genome database. Our initial search yielded one result, PF3D7_0807400, a 23kDa protein containing this conserved domain (residues 42–175 match the START-like domain signature IPR023393/G3DSA:3.30.530.20 with an E-value of 3.4E-23). A BLAST search of this protein against other protein databases, including that of *Saccharomyces*, retrieved a partial sequence alignment with ScCoq10 having 27% identity and 46% similarity. Comparison of the full length protein sequences in a multiple sequence alignment (Fig 1) yields a sequence identity of 19% with *S*. *cerevisiae* Coq10p and 21% with the *S*. *pombe* homolog, suggesting distant homology between PF3D7_0807400 and these proteins (compared to 23% identity between the human and *S*. *cerevisiae* Coq10p sequences and 29% identity between the human and *S*. *pombe* sequences). In addition, the *Plasmodium* protein shares several highly conserved critical residues for ubiquinone binding, as identified in *S*. *pombe* [5], including phenylalanine 68 and proline 70 (Fig 1). Mutation of these residues in the *S*. *pombe* protein caused a significant reduction in ubiquinone binding [5].

### PF3D7_0807400 complements *Sccoq10*

**Fig 2:**
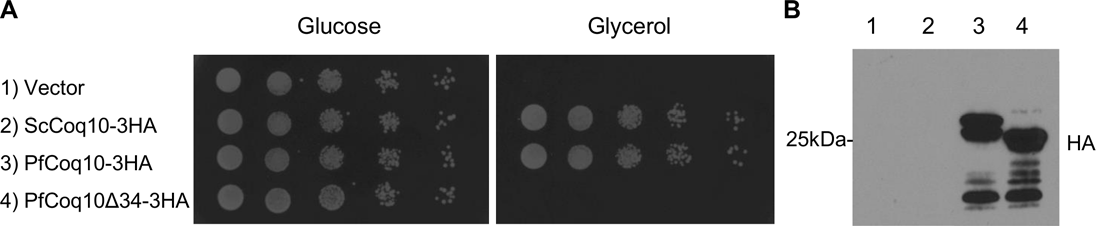
PF3D7_0807400 complements *Sccoq10*. (A) Complementation of *coq10*::*Kan* with a single copy plasmid (1–2) or a high copy plasmid expressing PfCoq10 variants from the ScTDH1 promoter. (B) Anti-HA Western blot of proteins extracted from the respective yeast strains.

Respiration deficiency caused by mutations in *ScCOQ10* can be complemented by several orthologues of the protein, including those of humans and the bacterium *Caulobacter crescentus* [3, 4], suggesting a functional conservation over long evolutionary distances. To test whether the *Plasmodium* protein would complement the respiration deficiency phenotype of the *Sccoq10*::*Kan* null allele, we synthesized a codon-optimized gene for expression in *Saccharomyces*. The synthetic gene was cloned into the high copy vector pRS426 that contained the strong constitutive *ScTDH1* promoter and a sequence encoding a carboxy-terminal 3HA epitope tag for detection. The mitochondrial targeting sequence prediction algorithm MitoProt [23] predicts a 34 amino acid targeting sequence for PF3D7_0807400, so a plasmid encoding PF3D7_0807400 with a truncation of the N-terminal 34 amino acids (Δ34) was constructed and transformed as well. ScCoq10 was likewise tagged and expressed in a single copy vector. All strains were viable on minimal media containing glucose as a carbon source, and western blotting confirmed the expression of the full length and Δ34 parasite proteins (**Fig 2B**), with anticipated sizes of 28kDa and 24kDa, respectively. ScCoq10p, expressed using its native promoter and cloned onto the centromeric vector pRS416, was not detected by Western analysis., but nevertheless complemented the respiratory defect. Remarkably, expression of the wild type parasite protein restored growth to a level comparable to that achieved by complementation with ScCoq10-3HA, suggesting that PF3D7_0807400 functionally complements the respiratory deficiency observed in the *coq10*::*Kan* strain (**Fig 2A**) and is properly localized to the mitochondria. This localization appears to be essential for complementation, as the strain expressing the truncated parasite protein exhibited the respiration deficient phenotype. Because of this ability to complement the ScCoq10-deficient line, we propose that PF3D7_0807400 is the *Plasmodium* orthologue of ScCoq10, PfCoq10.

### Expression and localization of PfCoq10 in *Plasmodium falciparum* parasites

**Fig 3:**
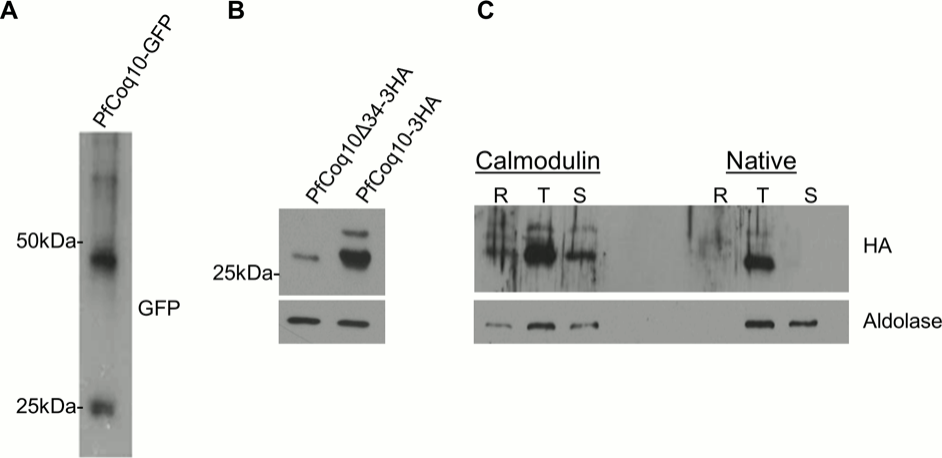
Expression of PfCoq10 in *Plasmodium falciparum* parasites. Western blots of saponin lysed parasite pellets from (A) PfCoq10-GFP expressing parasites, and (B) PfCoq10-3HA and PfCoq10Δ34-3HA expressing parasites. Aldolase was used as a loading control. (C) Comparison of PfCoq10-3HA expression levels expressed under control of either the calmodulin or the native promoter.

In order to detect PfCoq10 in *P*. *falciparum* parasites, we expressed tagged PfCoq10 fusion proteins using a bacteriophage recombination system in 3D7attB parasites [16]. We tagged PfCoq10 with a 3HA epitope or GFP and utilized the *P*. *falciparum* calmodulin promoter for robust expression. A control plasmid expressing PfCOQ10Δ34-3HA was also transfected. Upon confirming integration at the attB site, western blotting showed strong expression of PfCoq10-GFP at its predicted size of 50kDa, with minimal cleavage of the GFP detected (**Fig 3A**). A weaker band detected around 27kDa may represent cleaved GFP. PfCoq10-3HA was detected as two bands, estimated to be 27kDa and 25kDa (**Fig 3B**), with the majority in the lower band, presumably indicative of mitochondria processing. PfCoq10∆34-3HA, although not expressed as robustly, was detected as a band at about the same size as processed PfCoq10 (**Fig 3B**). Also, we expressed PfCoq10 under its native promoter at the attB site to determine its native level of expression. PfCoq10 was predominately detected in the trophozoite stage in both native and overexpressing parasite strains (**Fig 3C**). Interestingly, the protein was not detected in the schizont stages when expressed under the native promoter, but was still present, albeit in a lower quantity, in overexpressing parasites, suggesting that the protein may be degraded in later stages of intraerythrocytic development.

**Fig 4:**
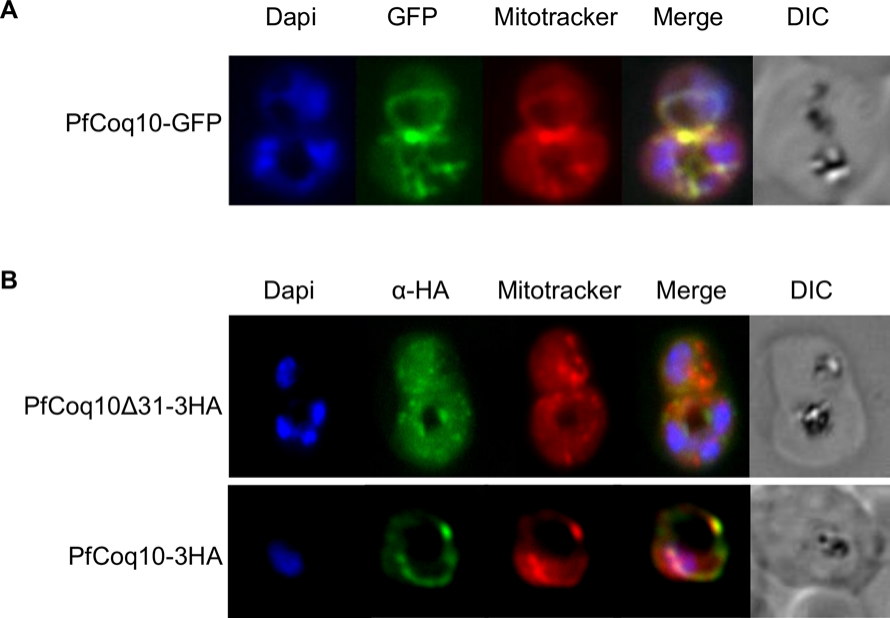
Localization of PfCoq10 to the mitochondria. (A) Live fluorescence microscopy of PfCoq10-GFP expressing parasites. (B) Immunofluorescence assay of parasites expressing HA-tagged PfCoq10 constructs.

The localization of PfCoq10 to the mitochondria appears to be crucial for complementation in yeast, as expression of Coq10Δ34 at comparable levels does not restore respiration. To confirm whether PfCoq10 is imported into the mitochondrion in parasites, we labeled PfCoq10-GFP parasites with MitoTracker, and assessed expression and localization in live parasites by fluorescence microscopy. Parasites showed strong expression of PfCoq10-GFP that colocalized with Mitotracker (**Fig 4A**). These results were further confirmed by immunofluorescence assays in parasites expressing PfCoq10-3HA. In contrast, PfCoq10Δ34 immunofluorescence showed mainly punctate staining in the cytoplasm, confirming that an N-terminal targeting sequence is required for mitochondrial localization (**Fig 4B**).

### PfCoq10 does not exhibit specific affinity for atovaquone

**Fig 5:**
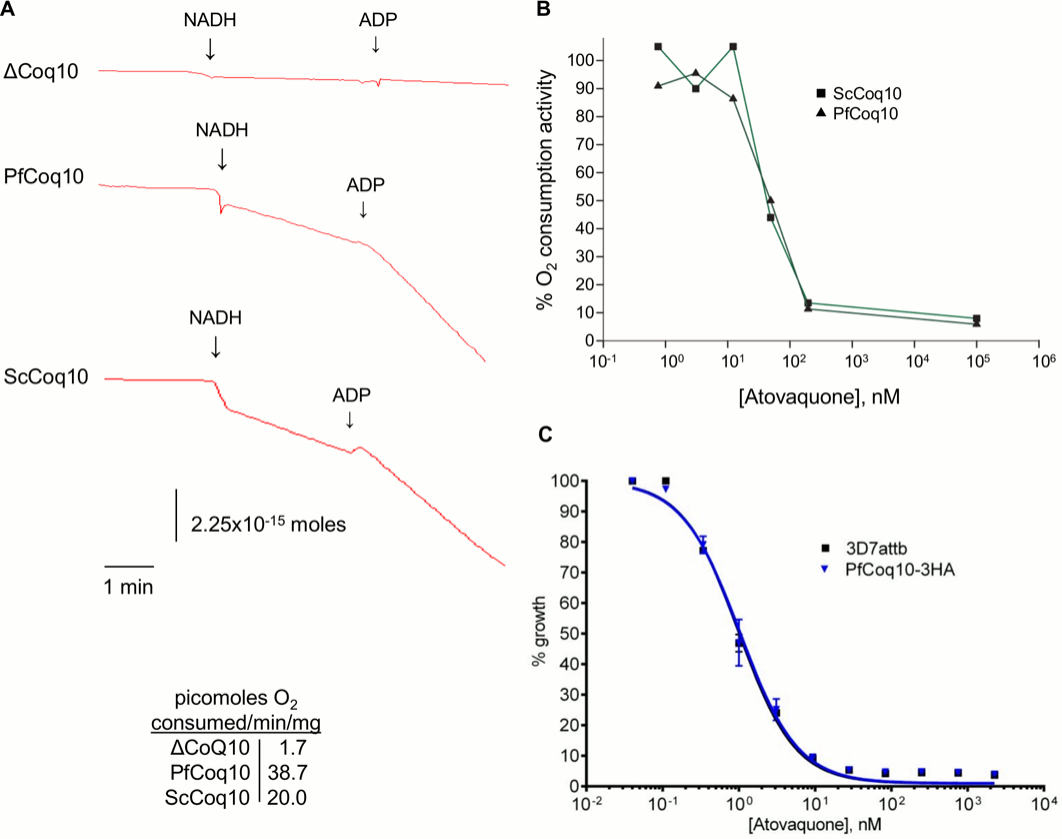
Sensitivity of PfCoq10 to atovaquone. (A) Measurement of O_2_ consumption in isolated yeast mitochondria, using NADH as the respiratory substrate. Addition of ADP stimulates respiration to its maximal rate by initiating rapid ATP synthesis. Representative reaction traces are shown. (B) Effect of increasing concentrations of atovaquone on respiration of mitochondria isolated from yeast expressing ScCoq10 or PfCoq10. (C) Growth inhibition of wild type 3D7attB and PfCoq10-3HA expressing parasites by increasing levels of atovaquone measured by the hypoxanthine incorporation assay.

The drug atovaquone is a validated antimalarial targeted against the cytochrome *bc*_*1*_ complex, where it competitively inhibits the ubiquinol oxidation by the enzyme [24, 25]. As atovaquone binds to the ubiquinol binding site of the bc1 complex [26, 27], we sought to determine if PfCoq10 is able to interact with atovaquone, potentially indicating that PfCoq10 serves as a chaperone, or even the delivery mechanism for the drug, as may be the case with CoQ. We hypothesized that parasites overexpressing PfCoq10 would increase the inhibitory concentration of the drug in the parasites due to sequestration of atovaquone by the excess protein. To investigate this, we analyzed the EC_50_ of atovaquone in PfCoq10-3HA expressing parasites by ^3^H-hypoxanthine incorporation; however, we found that the EC_50_ in these parasites was comparable to that in untransfected control parasites. This suggests that an excess of PfCoq10 does not alter atovaquone sensitivity in *P*. *falciparum* (**Fig 5C**). To further address this hypothesis, we isolated mitoplasts from the *Saccharomyces cerevisiae coq10*::*Kan* strain, and the strain complemented with either *ScCOQ10* or *PfCOQ10*. Initial respirometry experiments revealed that the *ScCOQ10* and *PfCOQ10* complemented strains had similar rate of respiration, as measured by NADH oxidase activity, while mitochondria from the *coq10*::*Kan* strain exhibited the expected inhibition in respiration (**Fig 5A**). We then treated these mitoplasts with atovaquone to determine if mitoplasts from the two complemented strains showed a differential inhibitory concentration, indicating that PfCoq10 has a higher affinity for atovaquone. However, no difference was seen in the mitoplast responses to atovaquone (**Fig 5B**). These findings suggest that, despite the evidence that PfCoq10 is involved in ubiquinone-binding, it does not have a functional interaction with the analog atovaquone.

## Discussion

Here we show the molecular genetic characterization of PfCoq10, the orthologue of ScCoq10, the second *Plasmodium* orthologue of the yeast ubiquinone synthesis pathway to be investigated. While this pathway is essential for cellular respiration in yeast, and CoQ likewise plays an essential role in parasites, very little is known about the synthesis and regulation of CoQ in parasites. As in its yeast counterpart, PfCoq10 contains the signature of a conserved START lipid binding domain and localizes primarily to the mitochondrion. Ubiquinone is utilized by the electron transfer complexes in the mitochondrial inner membrane, and analysis of the subcellular localization of the ScCoq10 protein revealed that it is also embedded within the same membrane [3]. We were unable to determine the sub-mitochondrial localization of PfCoq10, but analysis of Western blots of solubilized parasite proteins suggested that PfCOQ10-3HA is properly targeted and processed by the mitochondrial import machinery, and thus is expected to be similarly situated at the inner membrane, although we cannot be certain that all the excess protein is correctly localized.

Several genomewide RNA expression studies have demonstrated the expression of PfCoq10 mRNA [28–30]. Across these studies, there is significant variation in the potential level of expression as determined by percentile but it appears that the expression of the gene peaks in the trophozoite/early schizont stages of growth. To date, proteomic studies have failed to detect peptides derived from PfCoq10. This may suggest that the overall protein expression of PfCoq10 is low during blood stage growth of the parasite. It is interesting to note that an examination of the isolated yeast mitochrondrial proteome during fermentative and respiratory growth failed to detect ScCoq10p, although some of the enzymes involved in ubiquinone biosynthesis were detected [31]. From our studies, we have demonstrated that PfCoq10 is predominately expressed in the trophozoite stage, indicating a precise developmental regulation of PfCoq10, whereby it appears to be actively degraded during schizogeny. However, as the 3HA-tagged Coq10 proteins are expressed in the presence of the endogenous levels of PfCoq10, we cannot rule out the possibility of this degradation could be due to overexpression in both transgenic lines. While little is known about mitochondrial protein regulation in the parasite, at least one mitochondrial protease complex, ClpQY, has been characterized [32], and we have some preliminary evidence that this quality control protease may be involved in the regulation of PfCoq10 (B. J. Jenkins, unpublished data).

PfCoq10 shares some sequence similarity with its yeast coq10 orthologues, including conservation of several residues critical for CoQ binding. PfCoq10’s ability to complement a *coq10* null allele in *S*. *cerevisiae* suggests that it retains the ability to bind ubiquinone. While ScCoq10 is essential for cellular respiration, it remains to be definitively determined whether PfCoq10 is likewise critical for the function of mitochondrial electron transport in *P*. *falciparum*.

CoQ analogues such as atovaquone function as antimalarials by inhibiting the cytochrome bc_1_ complex of the parasite. It remains to be determined how such a lipophilic molecule is successfully trafficked through three aqueous spaces to the parasite mitochondrial inner-membrane. We hypothesized that PfCoq10 may have binding specificity for atovaquone as well as ubiquinone, and could therefore serve as a chaperone for the drug, possibly even as its mechanism of deliver to the respiratory complexes. However, parasites overexpressing PfCoq10 showed no shift in atovaquone sensitivity, as might be expected if it bound the drug. An alternate approach comparing respiration of isolated yeast mitochondria expressing either ScCoq10 or PfCoq10 showed no difference in sensitivity to atovaquone. This indicates that PfCoq10 may have a stringent specificity for ubiquinone, and as ubiquinone is essential to pyrimidine biosynthesis and mtETC [10], further analysis of PfCoq10 should provide a means of exploring aspects of these pathways.

## Supporting Information

S1 Appendix Primers used in this study. (DOCX)
