# Supplementary material for: Characterization of a Plasmodium falciparum Orthologue of the Yeast Ubiquinone-Binding Protein, Coq10p
Source: PLoS One. 2016 Mar 25;11(3):e0152197. doi: 10.1371/journal.pone.0152197 (PMC4807763; doi:10.1371/journal.pone.0152197)
Supplement: S1 Appendix — (DOCX) [file pone.0152197.s001.docx]

PF-10-5AV

5’-CTAcctaggtgataaatgaagattaatatatt-3’

PF-10-3BSI

5’-CAAcgtacgttttatcaagtgtaaaacgtccgtg-3’

pf-10-5ap

5’-ttttgggcccatattatgtgtccatatatattgtatgc-3’

pf-10-3av

5’-cattcctaggcatatcaggacaaaacaaaaaaataaataaaaatg-3’

PF-Δ34B

5’-ccaggatccatgagctttattaaaaccaacgatattgtg-3’

SC-5S

5’-gatGAGctccaacacttcttaatactgagc-3’

SC-3BSI

5’-caacgtacgcggagagccttctttagaagaaggttttag-3’
